# Is automated brachial cuff measurement of arterial pressure less accurate in cases of arrhythmia?

**DOI:** 10.1186/cc12114

**Published:** 2013-03-19

**Authors:** K Lakhal, S Faiz, M Martin, AS Crouzet, F Reminiac, S Ehrmann, R Cinotti, X Capdevila, K Asehnoune, Y Blanloeil, B Rozec, T Boulain

**Affiliations:** 1 Centre Hospitalier Universitaire Laennec, Nantes, France; 2 Centre Hospitalier Universitaire Bretonneau, Tours, France; 3 Centre Hospitalier Universitaire Lapeyronie, Montpellier, France; 4 Centre Hospitalier Universitaire Hotel-Dieu, Nantes, France

## Introduction

In cases of arrhythmia, the beat-to-beat variation of arterial pressure (AP) may impair the accuracy of automated cuff measurements. Indeed, this oscillometric device relies on the detection of arterial wall oscillations. Our aim was to determine, in ICU patients, whether brachial cuff measurements are really less reliable during arrhythmia than during regular rhythm.

## Methods

Patients with arrhythmia and carrying an intra-arterial catheter were prospectively and consecutively included in this multicenter study. After each arrhythmic inclusion, a regular rhythm patient was included. A second inclusion was possible in case of change in the cardiac rhythm. Three pairs of invasive and brachial cuff (Philips^® ^MP70 monitor) measurements of mean arterial pressure (MAP) were respectively averaged. Some patients underwent a second set of measurements, after a cardiovascular intervention (passive leg raising, volume expansion, initiation of/increase in catecholamine infusion) allowing the assessment of MAP changes.

## Results

In the 111 analyzed inclusions (in 103 patients) there was only one failure in displaying a brachial cuff measurement of MAP. Arrhythmic patients (atrial fibrillation 88%, frequent extrasystoles 7%, flutter 5%) were similar (*P >*0.3) to patients in regular rhythm for MAP (median 74 (IQR 67 to 80) vs. 75 (69 to 84) mmHg), SAPS II score, BMI, arm circumference, norepinephrine administration (36% vs. 35%), mechanical ventilation (80% vs. 81%), and site of the intraarterial catheter (radial artery: 80% vs. 85%). Between arrhythmic and regular rhythm patients: the agreement (Bland-Altman analysis) between invasive and brachial cuff measurements of MAP was similar (mean bias -0.4 ± 7.2 (limits of agreement -14/14) mmHg vs. 3.0 ± 8.2 (-13/19) mmHg, respectively); the detection of hypotension (invasive MAP <65 mmHg) by the brachial cuff was of similar reliability (area under the ROC curve (AUC) = 0.91 (95% CI = 0.80 to 0.97) vs. AUC = 0.98 (0.89 to 1), *P *= 0.2); and the detection of a response (>10% increase in MAP) to therapy was of similar reliability (AUC = 1 (0.85 to 1) (*n = *22) vs. AUC = 0.99 (0.78 to 1) (*n = *17), *P *= 0.5).

## Conclusion

These preliminary results suggest that arrhythmia does not impair the reliability of automated cuff measurements of MAP.

